# A Tenebrionid beetle’s dataset (Coleoptera, Tenebrionidae) from Peninsula Valdés (Chubut, Argentina)

**DOI:** 10.3897/zookeys.364.4761

**Published:** 2013-12-18

**Authors:** Germán H. Cheli, Gustavo E. Flores, Nicolás Martínez Román, Darío Podestá, Renato Mazzanti, Lidia Miyashiro

**Affiliations:** 1 CENPAT-CONICET, Bvd. Brown 2915, U9120ACF, Puerto Madryn, Argentina; 2 Laboratorio de Entomología, Instituto Argentino de Investigaciones de las Zonas Áridas (IADIZA, CCT-CONICET Mendoza), Casilla de correo 507, 5500, Mendoza, Argentina; 3 CENPAT-CONICET, Boulevard Brown num. 2915, U9120ACF, PUERTO MADRYN, Argentina

**Keywords:** Patagonia, Peninsula Valdés, Tenebrionidae, Pimeliinae, Tenebrioninae, Lagriinae, Edrotini, Nycteliini, Epitragini, Stenosini, Scotobiini, Opatrini, Belopini, *Blapstinus punctulatus*, *Ecnomoderes bruchi*, *Emmallodera hirtipes*, *Epipedonota cristallisata*, *Hylithus tentyroides*, *Leptynoderes strangulata*, *Leptynoderes tuberculata*, *Mitragenius araneiformis*, *Nyctelia nodosa*, *Rhypasma quadricollis*, Epitragus spp.

## Abstract

The Natural Protected Area Peninsula Valdés, located in Northeastern Patagonia, is one of the largest conservation units of arid lands in Argentina. Although this area has been in the UNESCO World Heritage List since 1999, it has been continually exposed to sheep grazing and cattle farming for more than a century which have had a negative impact on the local environment. Our aim is to describe the first dataset of tenebrionid beetle species living in Peninsula Valdés and their relationship to sheep grazing. The dataset contains 118 records on 11 species and 198 adult individuals collected. Beetles were collected using pitfall traps in the two major environmental units of Peninsula Valdés, taking into account grazing intensities over a three year time frame from 2005–2007. The Data quality was enhanced following the best practices suggested in the literature during the digitalization and geo-referencing processes. Moreover, identification of specimens and current accurate spelling of scientific names were reviewed. Finally, post-validation processes using DarwinTest software were applied. Specimens have been deposited at Entomological Collection of the Centro Nacional Patagónico (CENPAT-CONICET). The dataset is part of the database of this collection and has been published on the internet through GBIF Integrated Publishing Toolkit (IPT) (http://data.gbif.org/datasets/resource/14669/). Furthermore, it is the first dataset for tenebrionid beetles of arid Patagonia available in GBIF database, and it is the first one based on a previously designed and standardized sampling to assess the interaction between these beetles and grazing in the area. The main purposes of this dataset are to ensure accessibility to data associated with Tenebrionidae specimens from Peninsula Valdés (Chubut, Argentina), also to contribute to GBIF with primary data about Patagonian tenebrionids and finally, to promote the Entomological Collection of Centro Nacional Patagónico (CENPAT-CONICET) and its associated biodiversity data. For these reasons, we believe that this information will certainly be useful for future faunistic, ecological, conservational and biogeographical studies.

## General description

**Purpose:** The general purpose of this dataset is to ensure accessibility to data associated with Tenebrionidae specimens from Peninsula Valdés (Chubut, Argentina) deposited in the Entomological Collection of Centro Nacional Patagónico (CENPAT-CONICET), Argentina. At present, datasets about Tenebrionidae beetles in GBIF portal contains only two records of Tenebrionids for whole Patagonia (accessed 04/13/2013), one of these is a fossil record, interpreted as Tenebrionidae indet (Locality: Rio Pichileufu, Rio Negro; Data Publisher: Marine Science Institute, UCSB; Dataset: Paleobiology Database; http://data.gbif.org/occurrences/40876235/). Taking into account this scenario, the dataset presented here makes a significant contribution of primary data about Patagonian tenebrionids. In addition, this information could be useful for future faunistic, ecological and conservation studies. Finally, through this dataset we intend to promote the Entomological Collection of Centro Nacional Patagónico (CENPAT-CONICET) and their associated biodiversity data.

## Project details

**Project title:** A Tenebrionid beetle’s dataset (Coleoptera, Tenebrionidae) from Peninsula Valdés (Chubut, Argentina)

**Personnel:** Germán H. Cheli (Resource creator, Collector, Tenebrionid identification, Curator, Metadata provider, Content provider); Gustavo E. Flores (Content provider, Tenebrionid identification); Nicolás Martínez Román (Collector, Processor, Data digitizer, Colection assistant); Darío Podestá (Processor, Collection assistant, Data digitizer); Renato Mazzanti (Programmer, Data base manager); Lidia Miyashiro (Programmer, Data base assistant).

**Funding:** This work was supported by Consejo Nacional de Investigaciones Científicas y Técnicas of Argentina (CONICET), including a PhD fellowship and the project: “*Estudios sistemáticos y biogeográficos de coleópteros epígeos de la estepa patagónica, con énfasis en la influencia de factores ambientales, linajes filogenéticos y patrones de especiaciónpara ser aplicados en la conservación de su diversidad*” (grant ref. PIP 112-201101-00987). Digitalization of this biological collection is supported by *Sistema Nacional de Datos Biológicos* (SNDB, MINCyT, Argentina) by the project “*Informatización, Conservación y Fortalecimiento de las Colecciones del Centro Nacional Patagónico- CONICET*” (grant ref. SNDB-F9).

**Study area descriptions/descriptor:** Peninsula Valdés is a wide plateau, extending 4,000 km^2^ in the NE of Chubut Province (42°05'–42°53'S, 63°35'–65°04'W). It is considered part of different biogeographic provinces by different authors, thus some include it in Patagonia (Soriano 1956, Morrone 2001, Morrone et al. 2002) while other authors consider it is in the Monte Phytogeographic Province (Cabrera and Willink 1973, Roig-Juñent and Flores 2001, Roig et al. 2009). The mean annual temperature in this area is 13.4°C, showing wide range during summer (Labraga et al. 2008). Predominant winds are from the western quadrant (Barros and Rodríguez Seró 1981) and annual rainfall ranges from 175 to 225 mm (Súnico et al. 1994).

Despite Peninsula Valdés is one of the largest arid areas included in Argentinian conservation programs, at present there is a fragmented knowledge of terrestrial arthropods (Cheli et al. 2010). Coleopterans are the most abundant and diverse non-social insects of Peninsula Valdés, and Tenebrionidae is the most numerous family among them (Cheli et al. 2010). These beetles play an important role as decomposers in arid lands (Flores 1998) and some species are omnivorous (Cheli et al. 2009). Moreover, tenebrionid beetles are sensitive indicators of biodiversity and habitat change (Cheli 2009).

**Figure 1.**
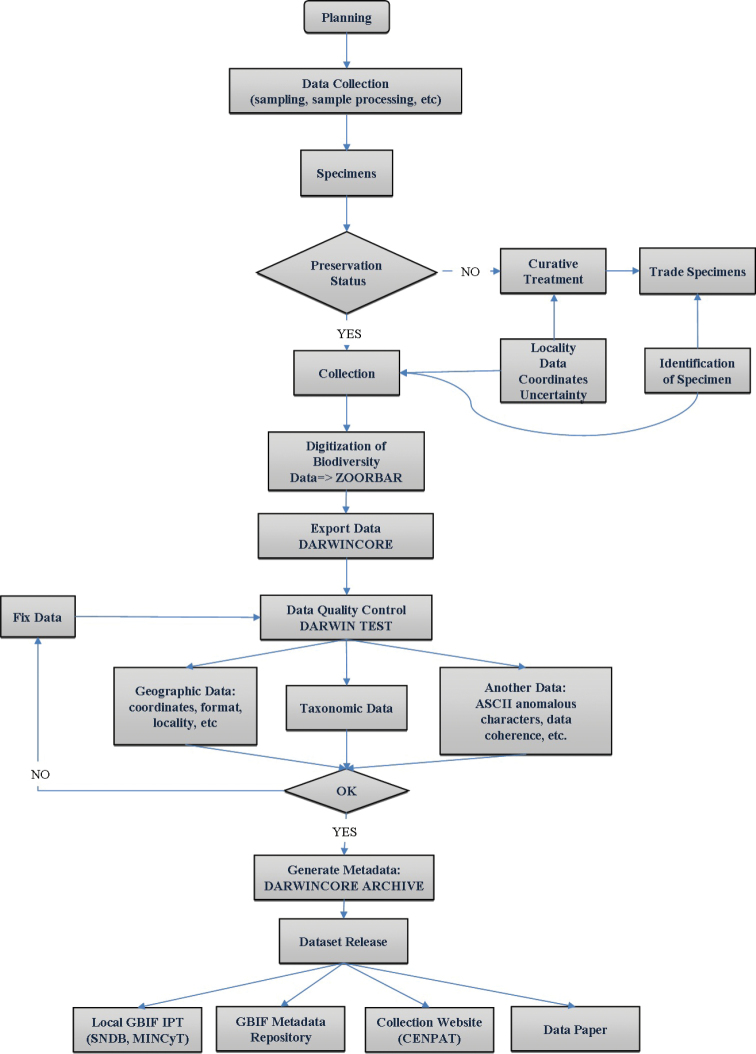
Flow chart describing the methods procedure: collection, digitalization and data publishing.

**Design description:** Samples were processed in the laboratory and adult tenebrionid specimens were obtained (Figure 1). Preservation status of individuals was examined and those showing original good curatorial condition were housed in the collection. Species determination was done following reviews and keys (Kulzer 1955, 1963, Flores 1997, 1999) and comparing the collected material with specimens housed at CENPAT-CONICET and IADIZA-CONICET entomological collections. The classification of Tenebrionidae to tribes and subfamilies was based on the one proposed by Bouchard et al. (2005). Taxonomical determination of problematic specimens was verified by PhD Gustavo Flores (IADIZA-CONICET), a taxonomist specialized in South American tenebrionid beetles. Thereafter, data associated with specimens were digitized using ZOORBAR software (http://www.gbif.es/zoorbar/zoorbar.php). Geo-referencing details and current accurate spelling of scientific names are fully described in the “Quality control description” section. The dataset was exported on DarwinCore v.1.4 (http://www.gbif.es/Recursos2.php), postvalidation was applied using DARWINTEST software (http://www.gbif.es/darwin_test/Darwin_Test_in.php) and the metadata was integrated to the dataset in DarwinCore Archive format. Finally, the dataset was provided to Sistema Nacional de Datos Biológicos, Ministerio de Ciencia, Tecnología e Innovación Productiva (SNDB, MinCyT, Argentina) and to the Global Biodiversity Information Facility (GBIF), by means of their Integrated Publishing Toolkit (IPT) (Figure 1).

**Data published through** GBIF: http://www.cenpat-conicet.gov.ar:8080/ipt-2.0.3/resource.do?r=cnp-e

## Taxonomic coverage

**Figure 2.**
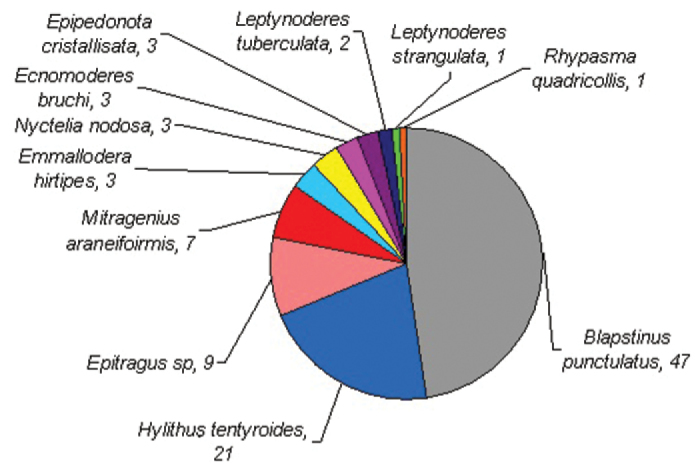
Distribution of tenebrionid species from Peninsula Valdés included in the dataset. The number next to the specific name indicates its percentage.

**Figure 3.**
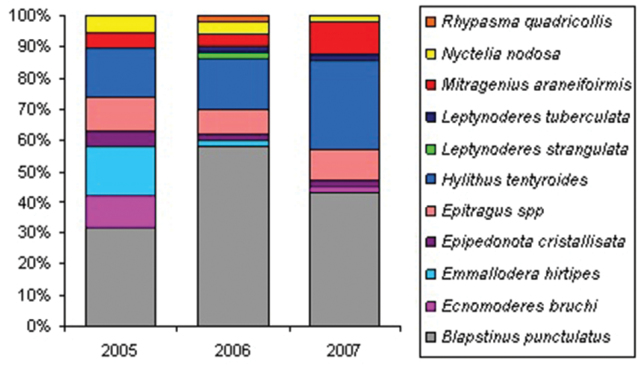
Distribution of tenebrionid species from Peninsula Valdés included in the dataset among the sampling years (2005 to 2007).

**General taxonomic coverage description:** Dataset comprise 3 subfamilies, 7 tribes and 11 species. The most representative subfamilies are Pimeliinae and Tenebrioninae, each depicting half of the records. At tribal taxonomical level, Pimeliinae is the richest one, including Edrotini (21.2%), Nycteliini (12.7%), Epitragini (9.3%) and Stenosini (2.5%). Tenebrioninae comprises only two tribes, Scotobiini (5.9%) and Opatrini (47.5%). While Lagriinae, the third subfamily found, has only one record (*Rhypasma quadricollis* Fairmaire, Belopini tribe (0.8%)). *Blapstinus punctulatus* Solier is the most common species of the dataset, including more than 30% of the records in each year and more than 50% considering the period sampled; follow in importance by *Hylithus tentyroides* Lacordaire (16% in 2005 and 2006) and *Emmallodera hirtipes* Kulzer (16% in 2007) (Figures 2 and 3).

## Taxonomic ranks

**Kingdom:** Animalia

**Phylum:** Arthropoda

**Subphylum:** Hexapoda

**Class:** Insecta

**Order:** Coleoptera

**Suborder:** Polyphaga

**Infraorder:** Cucujiformia

**Superfamily:** Tenebrionoidea

**Family:** Tenebrionidae

**Subfamily:** Lagriinae, Pimeliinae, Tenebrioninae

**Tribe:** Belopini, Edrotini, Epitragini, Nycteliini, Stenosini, Opatrini

**Genus:** Epitragus, Rhypasma, Hylithus, Epipedonota, Mitragenius, Nyctelia, Ecnomoderes, Blapstinus, Emmallodera, Leptynoderes

**Species:** *Rhypasma quadricollis*, *Hylithus tentyroides*, *Epipedonota cristallisata*, *Mitragenius araneifoirmis*, *Nyctelia nodosa*, *Ecnomoderes bruchi*, *Blapstinus punctulatus*, *Emmallodera hirtipes*, *Leptynoderes strangulata*, *Leptynoderes tuberculata*

**Common names:** darkling beetles, insect, beetles

## Spatial coverage

**Figure 4.**
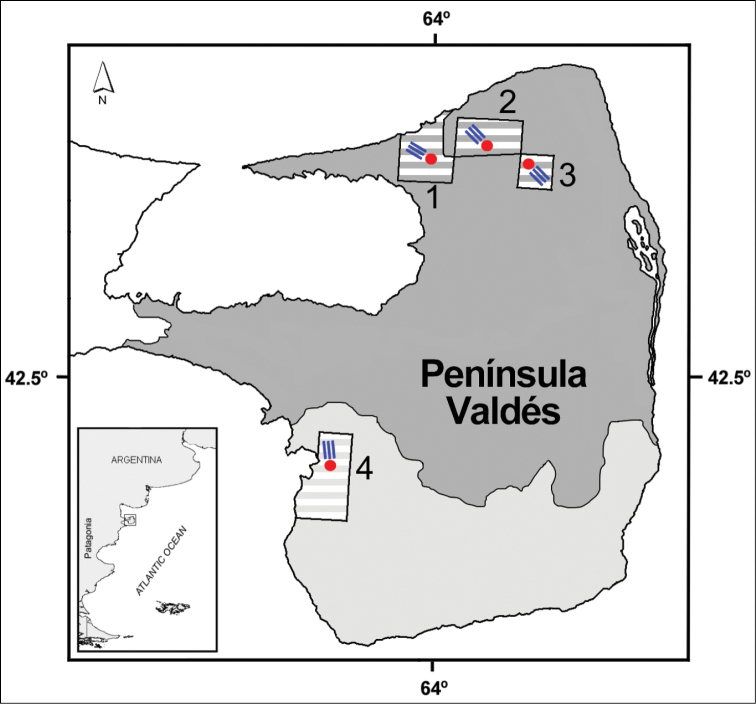
Location of the sampled farms (striped squares: **1** El Progreso **2** El Centro **3** La Falsa **4** San Pablo de Valdés). Colored areas show the main physiognomy units of Peninsula Valdés, shrub steppe (gray) and herbaceous steppe (white). Blue lines and red circles indicate sampling transects and water wells, respectively.

**General spatial coverage:** The Natural Protected Area Peninsula Valdés (Figure 4) is located on the Atlantic coast of Chubut province (Argentina) and was declared a World Heritage site by UNESCO in 1999.

Physiographycally the peninsula is characterized by a flat landscape with three endorheic depressions (Salina Grande, Salina Chica, and Gran Salitral) with ephemeral hypersaline lakes. There are no permanent watercourses in the area and due to the narrow isthmus connecting peninsula and continent, allochthonous courses cannot gain access (Beltramone 1983, Alvarez et al. 2010). Geologically, Peninsula Valdés is formed by Oligo-Miocenic marine sediments and exhibits a continuous cover of aeolian sediments intermingled with quaternary gravels (Súnico et al. 1994, Haller et al. 2001). The actual landscape configuration of the region was caused by Pre-Quaternary intense tectonic movements and strong periglacial winds during Pleistocene period (~1myrs). In general, soils correspond to the Aridisol and Entisol orders (Rostagno 1981).

Peninsula Valdés entails great importance from a biological perspective (UNESCO 1999, Yorio et al. 2005, Cheli et al. 2010). Floristically about 130 species of plants are found in the region, while faunistically it supports an important vertebrate biodiversity: 13 species of reptiles, 108 of terrestrial birds (Plan de Manejo del Área Protegida Sistema Península Valdés 1998) and 28 of terrestrial mammals (Nabte et al. 2009). It is interersting to point out that terrestrial arthropods show the greatest diversity, with about 160 species included in 18 orders and 52 families (Cheli et al. 2010). Nevertheless, the knowledge of terrestrial fauna is still fragmentary for this area (Nabte et al. 2009, Cheli et al. 2010).

Nowadays, human population in Peninsula Valdés is scarce, including Puerto Pirámides as the only urban center, a few settlers dispersed among farms and temporary artisanal fishing camps. Since 1882 the economy of the region has been based on sheep livestock (Barba Ruíz 2003). In general, grazing is practiced extensively in big paddocks (more than 2,500 ha) with a single permanent water point. At present, there are an estimated number of 90 sheep farms and 80,000 sheep in Peninsula Valdés (Baldi et al. 1997). Furthermore, during the last two decades, tourism activity has increased significantly, with 250,000 tourists visiting the area each year (Nabte et al. 2009).

Peninsula Valdés shows serious signs of deterioration caused by human activities. Nearly 90% of its natural grasslands are in a poor state of conservation with soils and vegetation severely degraded by overgrazing. Even though the impact that land use and touristic activities caused on terrestrial vertebrates has not been evaluated (Nabte et al. 2009), it is known that terrestrial arthropods have shown significant changes as a consequence of sheep overgrazing (Cheli 2009). This feature allowed considering them as biological indicators of natural environment disturbance (Cheli et al. 2010).

Finally, even though Peninsula Valdés has been the target of several scientific contributions, their biogeographical identity is still a conflictive issue. Therefore, this data set improves the knowledge of the tenebrionids of the area and it could be useful to clarify the biogeographical identity of the peninsula.

### Coordinates

43°5'24"S and 41°55'48"S Latitude; 64°52'12'"W and 63°23'60"W Longitude.

### Temporal coverage

February (mid-summer in the Southern hemisphere), years 2005-2006-2007.

## Natural collections description

**Parent collection identifier:** CNP

**Collection name:** Colección Entomológica del Centro Nacional Patagónico “Francisco Pascasio Moreno”

**Collection identifier:** CNP-CE

**Specimen preservation method:** All specimens are preserved in 70% ethyl alcohol. Individuals were stored in eppendorfs (1.5ml) or jars (20ml) full of alcohol (70%). All specimens belonging to the same species, in good curatorial conditions and found in the same sample (same date and site), were considered as a lot. Lots are the curatorial units of the collection. Each one contains among 1 to 10 specimens and have a unique collections’ number assigned (catalog number). In those cases where the lot had more than one eppendorf or jar, all of them were kept into a Ziploc© plastic bag and then located into a hermetic bigger jar filled with alcohol (70%). Each specimen was accompanied by its original label and a new one stating their unique catalog number, both labels were placed within the eppendorf or jar. If the genitalia of some specimen was studied, it was conserved into a different eppendorf inside the Ziploc© bag that contains the exemplar. All jars are kept in a room without windows at a relatively constant temperature (18°C).

Fluctuations in the temperature and relative humidity levels can be the biggest cause of environmental damage to biological collections (Alten 1999). In this sense, the use of alcohol for conserving entomological material helps to control the harmful effects of the factors mentioned above. Moreover, the best preservative for alcoholic collection of small invertebrates is 70% ethyl alcohol (Levi 1966). In addition, for insect DNA preservation the highest yields and least sheared DNA were obtained from specimens preserved in ethanol. Whereas DNA from individuals conserved in other type of alcohol was degraded to small fragments and dried pinned specimens gave undetectable yields of DNA (Post et al. 1993). Finally, when specimens are preserved in alcohol, they conserve their joints soft, thus greatly reducing the likelihood of damage during handling.

**Curatorial unit:** 118 (with an uncertainty of 0).

## Methods

**Figure 5.**
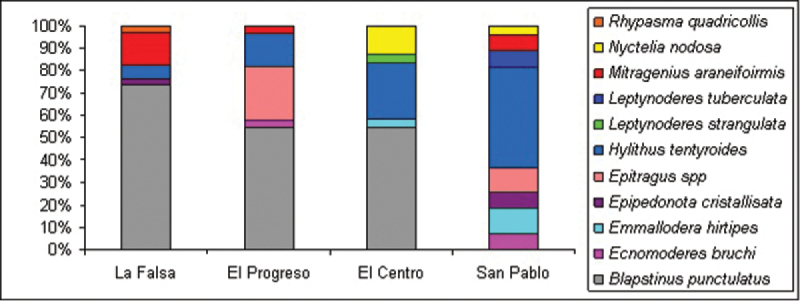
Distribution of Tenebrionid species among the sampled farms. Note that El Progreso, El Centro and La Falsa, belong to the shrub steppe physiognomy unit while San Pablo de Valdés, to the herbaceous steppe.

**Figure 6.**
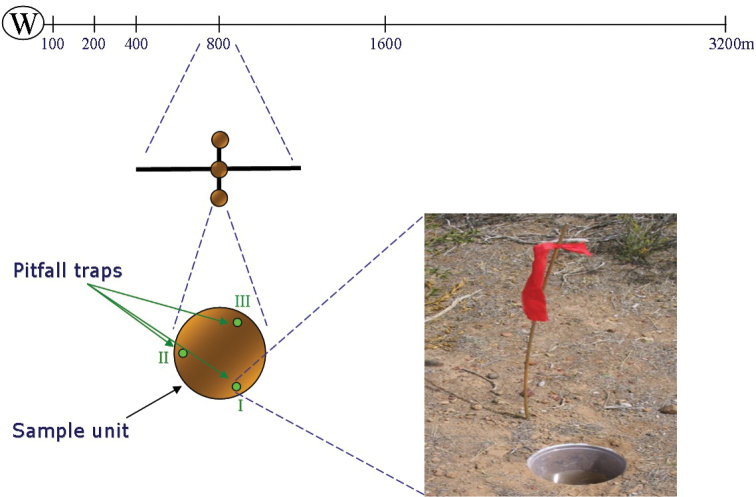
Design of sampling method. Each transect (3 per farm) consist of 6 sampling sites along a gradient of grazing disturbance (100, 200, 400, 800, 1600, and 3200m from water well). Each sample unit consists of 3 pitfall traps.

**Method step description:** Figure 1 summarizes the methodological procedure. *Planning and data collection*: The dataset was obtained from PhD thesis of G.H. Cheli (2009) whose main objectives were to improve the knowledge of the epigeal arthropods living in Peninsula Valdés and to study the effect of grazing on this group of animals in the region. This was the first study carried out in the area that used pitfall traps, for this reason the art of capture should be optimized (see Cheli and Corley 2010). Due to strong water limitations in Peninsula Valdés, grazing intensity varies in relation to the water well proximity (Lange 1969). This gradient of disturbance offers an experimental opportunity to study the effects of grazing over artropodofauna avoiding the methodological problems associated with other experimental designs (see Andrew 1988, James et al. 1999). Therefore, the grazing impact on terrestrial arthropods of Peninsula Valdés was assessed through transects related to water wells (Figures 5 and 6) (see "Sampling description"). *Data curation*: Damaged specimens were excluded from the dataset. When necessary, curative treatment was provided and these individuals were reserved like trade specimens. *Identification*: The taxonomic identification was carried out in the laboratory using suitable literature (see details in the “Design description” section). *Data management*: Biodiversity data existing on the specimens’ labels (i.e. collection code, catalog number, species identification, name of determiner, locality, collection date, habitat, altitude, GPS coordinates, collector, ecological observations and notes) were included in a digital database using ZOORBAR software (http://www.gbif.es/zoorbar/zoorbar.php). Data were exported in Darwin Core (v1.4) format. *Data quality enhancement*: see details in the section on quality control. *Data publishing*: Once postvalidation was applied, dataset was transformed into DarwinCore Archive format associating their metadata. Finally, the dataset was published into the Global Biodiversity Information Facility (GBIF) portal, by means of their Integrated Publishing Toolkit (IPT) and provided to Sistema Nacional de Datos Biológicos, Ministerio de Ciencia, Tecnología e Innovación Productiva (SNDB, MinCyT, Argentina).

**Study extent description:** The variety of soils and plant communities living in the region determines the presence of several types of habitats in Peninsula Valdés. In the north portion, the dominant physiognomy is a shrub steppe of *Chuquiraga avellanedae*, *Chuquiraga histrix*, *Condalia microphylla*, *Lycium chilense*, *Schinus polygamous* and *Prosopidastrum globosum*, accompanied by the grasses *Nassella tenuis*, *Piptochaetium napostaense* and *Poa ligularis* (Bertiller et al. 1981) (Figure 4). In the south, the shrub steppe is replaced by a herbaceous steppe where *Sporobolus rigens* becomes the most important species along with patches of *Chuquiraga avellanedae* and *Hyalis argentea* (Bertiller et al. 1981) (Figure 4). The dataset presented here comprise tenebrionid beetles sampled in both physiognomy units, with three sampling sites (farms) in the shrub steppe and one in the herbaceous steppe (Figure 4). Sampling was made during the middle austral summer (February) of 2005, 2006 and 2007. Dataset include specimens from sampling sites along a gradient of grazing disturbance. This dataset also shows that several entomofaunal differences between these two main ecological areas of Peninsula Valdés are evident when the North and South collecting sites are taken into account separately. The main variation is observed in dominant tenebrionid species: *Blapstinus punctulatus* is the most common species in the northern part of Peninsula Valdés, while *Hylithus tentyroides* dominates in the southern one (Figure 5).

**Sampling description:** The specimens composing this dataset were collected using pitfall traps. This trapping technique was selected for several reasons: 1- it is the most frequently used method for sampling ground-dwelling arthropods (Niemelä et al. 1992, Pekár 2002, Phillips and Cobb 2005); 2- pitfall traps serve to evaluate the distribution of macroinvertebrates in diverse ecosystems at different scales, also to describe activity patterns and habitat associations, as well as establishing the effects of disturbances on biodiversity (Niemelä et al. 1992, Pekár 2002, Mazía et al. 2006); 3- in some cases, pitfall traps are the only alternative for sampling arthropods (Niemelä et al. 1993, Pearsal 2007); 4- their objectivity is a crucial feature that allows better comparisons (Vennila and Rajagopal 1999); 5- pitfall traps are a quick and cheap method to capture arthropods.

Four sheep farms, with a single well per fenced plot, were selected for conducting the study (three in the northern shrub steppe and one in the southern herbaceous steppe) (Figure 4). The sampling design consisted on 3 transects per farm covering different grazing intensities in relation to the position of the water well (Figures 4 and 6). At each transect, six sampling sites varying in distance to the water well were established (100, 200, 400, 800, 1600 and 3200m) (Figure 6). Three pitfall traps were placed at each sampling site and then treated as a sample unit. A total of 12 transects with 216 traps per year were established (making 648 traps in three years).

In order to enhance catches, each trap was placed in vegetation patches and neatly buried in the soil near bushes. Traps consisted of plastic jars of 12cm in diameter at the opening and 12cm deep. The quantity of traps used guaranteed capturing almost all taxa dwelling in the area (Cheli and Corley 2010). Besides, the type of traps employed has proved to be the most efficient pitfall configuration for this region. Each trap was filled with 300ml of a 30% solution of ethylene glycol used as preservative and opened on-site for two weeks (Cheli and Corley 2010).

**Quality control description:** Following Wieczorek (2001) and Chapman and Wieczorek (2006), validation of geographic, taxonomic and additional data was incorporated in the digitalization process at several steps (Figure 1), as well as the geo-referencing of all specimens. Therefore, the geographic coordinates were recorded in decimal degrees using a Garmin eTrex Legend GPS (WGS84 Datum) with an accuracy of less than 10 m and with at least 5 satellites. The calculated uncertainty was 2.83 meters (Wieczorek 2001). In addition, the geo-coordinates of each specimen were verified using digital cartography (satellite images; Quantum GIS v1.7; Google Earth). The taxonomical identification of specimens, scientific names and their current accurate spelling were reviewed using suitable literature (Kulzer 1955, 1963, Flores 1997, 1999) and verified by a tenebrionid’s specialist (G. E. Flores). Other postvalidation procedures (including geographic coordinate format, coordinates within country/provincial boundaries, congruence between collection and identification dates absence of ASCII anomalous characters) were checked by use of the Darwin test software (http://www.gbif.es/darwin_test/Darwin_Test_in.php).

## Dataset

### Dataset description

**Object name:** Darwin Core Archive A Tenebrionid beetle’s dataset (Coleoptera, Tenebrionidae) from Peninsula Valdés (Chubut, Argentina)

**Character encoding:** UTF-8

**Format name:** Darwin Core Archive format

**Format version:** 1.0

**Distribution:** http://www.cenpat-conicet.gov.ar:8080/ipt-2.0.3/archive.do?r=cnp-e

**Publication date of data:** 2013-01-09

**Language:** English

**Licenses of use:** This work is licensed under a Creative Commons CCZero 1.0 License http://creativecommons.org/publicdomain/zero/1.0/legalcode

### External datasets
Dataset description

**Object name:** Centro Nacional Patagónico (CENPAT-CONICET)

**Distribution:** http://www.cenpat-conicet.gov.ar:8080/ipt-2.0.3/archive.do?r=cnp-e

### Dataset description

**Object name:** Ministerio de Ciencia y Tecnología de Argentina (Sistema Nacional de Datos Biológicos - SNDB)

**Distribution:** http://datos.sndb.mincyt.gob.ar/portal/datasets/resource/162

**Metadata language:** English

**Date of metadata creation:** 2013-01-09

**Hierarchy level:** Dataset
